# Cardiovascular Disease Training Programmes: Three Schemes to Train Leaders for Future Challenges

**DOI:** 10.5334/gh.1361

**Published:** 2024-10-07

**Authors:** Amitava Banerjee, Dorairaj Prabhakaran, Kay-Tee Khaw, Marie Chan Sun, Vilma Irazola, Goodarz Danaei, Pablo Perel

**Affiliations:** 1 Institute of Health Informatics, University College London, London, UK; 2 Barts Health NHS Trust, London, UK; 3 Public Health Foundation of India, Delhi, India; 4 MRC Epidemiology Unit, University of Cambridge, Cambridge, UK; 5 University of Mauritius, Reduit, Mauritius; 6 South American Center of Excellence for Cardiovascular Health, University of Buenos Aires, Buenos Aires, Argentina; 7 Harvard Chan School of Public Health, Harvard University, Boston, USA; 8 Department for Non-Communicable Diseases, London School of Hygiene and Tropical Medicine, London, UK

**Keywords:** cardiovascular, training, epidemiology, research, implementation, capacity

## Abstract

Cardiovascular disease (CVD) represents the largest burden of disease globally and despite the availability of strong evidence supporting cost-effective treatments for people with CVD, the implementation of these treatments remains low, especially in low-income settings. Shortages in workforce have led to focus on how to increase clinical capacity. However, a simplistic focus on training clinicians will not fill the gaps in research, policy and implementation, which also need to be addressed at the same time. There are multiple efforts to develop early career capacity across diverse areas at national and international level to address these gaps. To-date, there have been limited efforts to compare or evaluate such programmes, and there are no efforts to harmonise such programmes to take advantage of synergies. We now compare three international programmes on global cardiovascular research to train individuals in their early- and mid-career by aims, experience and outputs.

## Introduction

The reality of the high and growing burden of cardiovascular disease (CVD) is well-recognised in terms of individuals, health systems and economies (1,2). Although the global CVD community has been highly successful in identifying the causes of CVD and, more importantly, in demonstrating strong evidence for cost-effective treatments, the implementation of these treatments remains notably low. This gap between evidence and care is especially pronounced in low- and middle-income countries (LMICs). Analogous to the so-called ‘inverse care law’ (11), the implementation gap is greatest and the lack of resource most striking where it is most needed, i.e., in low-middle income settings. Sub-Saharan Africa has particular disparities by knowledge and evidence generation, dissemination and implementation (3,4,12,13). Moreover, global comparative studies including LMICs are relatively rare. In LMICs, especially Sub-Saharan Africa, more epidemiologic research regarding CVD, whether aetiology, incidence or prognosis, is urgently needed to provide context-specific recommendations.

Across countries, there is an urgent need to put into practice what we know, with multiple barriers at system levels from inequalities in access to care to representation in clinical trials (3,4,5,6). Despite multiple international initiatives to address these issues in both CVD-specific and general policies, including the World Health Organization’s ‘25 × 25’ goal to reduce risk of premature (age < 70 years) mortality from noncommunicable, chronic diseases, including CVD, by 25% by 2025, major gaps still remain (7). Improving CVD requires strengthening practice and policy at global levels, but also importantly it requires strengthening in global cardiovascular research (4,8,9,10).

The need for global approaches to training and capacity-building in CVD treatment and prevention has been recognised for many years (14), and there are a plethora of regional, national and international mentoring and training schemes in CVD globally. However, there are three training programmes that are somehow unique and related, as all of them are global in their ambition, they are addressed to early- and mid-career and have a focus on research in LMICs and on equity. These programmes are the World Heart Federation Emerging Leaders Programme (7,15), the Ten-day International Teaching Seminar on Cardiovascular Disease Epidemiology and Prevention (16,17), and the Bernard Lown Scholars in Cardiovascular Health Programme (18). There may be potential opportunities to improve the cross-talk and the inter-relationships between these programmes. We therefore review three training schemes to contribute to global CVD prevention to better understand opportunities to work across schemes. Table 1 highlights key features of the three programmes and shows similarities and differences.

**Table 1:** Comparison of three international early career CVD training programmes.


	WHF EL	10-DAY ISCEP	LOWN SCHOLARS PROGRAMME

Year started	2014	1968	2008

Number of participants per year	25	36–40	8–10

Total number of trained participants	221	1800	104

Number of countries represented	53	100	23

% Female	46	40	44

% LMIC	52	60	100

% Sub-Saharan Africa	20	15	Not specified

Focus	CVD implementation science and policy	10-day residential training programme	CVD prevention research

Format	5-day residential seminar with 1-year seed-funded group project	Fellows become active in CVD prevention research, implementation and policy	4-week residential Summer Programme, chance to apply for 1–2 year seed funding, annual appointment as visiting scholars renewed each year

Outputs/expectations		Collaborative international research programs	Collaborative research and implementation projects on CVD prevention with potential for scale-up

Ongoing networking/collaboration	Involvement of Emerging Leaders in World Heart Federation activities, including Roadmaps, Committees, White Papers and the Emerging Leaders scheme itself.	10-day residential training programme	14 teams created within the Programme and used to recruit new Scholars and to develop ideas and proposals for funding. Monthly town-halls and Discussion series.


## The International Society of Cardiovascular Disease Epidemiology and Prevention (ISCEP) Ten-Day International Teaching Seminar on Cardiovascular Disease Epidemiology and Prevention

The seminars were founded in 1968 by Professors Jerry and Rose Stamler and Richard Remington from the USA, and Professor Geoffrey Rose from the UK, with support from the International Society and Federation of Cardiology (since 1998, World Heart Federation), and Council on Epidemiology and Prevention and from 2008, the International Society of Cardiovascular Disease Epidemiology and Prevention. The main aim of the seminar was and is to increase the body of people around the world who have the needed skills to carry out epidemiologic studies and to strengthen the efforts to prevent mass CVD. The founders recognised the need for training in research, and also the constraints on time and resources, limiting the ability of workers in low- and middle- income countries to obtain this. They thus developed a ten-day course to provide basic training. The basic training is in fundamental epidemiologic principles and methods and biostatistics with focus and practical examples on CVD epidemiology and prevention. Approximately 36 fellows are accepted from international applications each year. Nominees should normally be at the postgraduate level with residency training or its equivalent and be interested in CVD epidemiology. Preference is given to younger candidates, with little or no formal training in epidemiology. Tuition, board and accommodation are provided without cost to fellows. To-date, over 1800 physicians and scientists from over 100 nations have been fellows of the seminar, and 52 seminars have now taken place in 39 different countries.

The success of the seminars is evidenced by the large numbers of past fellows who are now active in the field, as leaders of research and public health programmes throughout the world. Many of the major achievements in CVD epidemiology and prevention have been made by past fellows who had their first introduction to the area at a seminar. Past fellows have also initiated national seminars based on the same model in many countries including the USA, Spain, Japan, Italy, Brazil, Venezuela and Thailand to further increase the impact.

A major focus of the seminar is the facilitation of exchange of scientific knowledge and expertise internationally and the fostering of collaborative research work. The activities stimulated by the seminar have generated a series of International Conferences in Preventive Cardiology. At each seminar, fellows and faculty discuss research and prevention programmes and learn from different international approaches of communities in all continents. The aims are not just the training of an international corps of persons working on the prevention of CVD but the making of bridges across countries and cultures through peaceful international scientific cooperation. More recent seminars have taken place in low- and middle-income countries to reflect the rising burden of CVD and need for capacity building in those regions.

This initiative comes about through the good-will of all those involved in the organization. The host country supports the costs of local organization, accommodation and board of the participants. The seminar faculty volunteer their efforts for the seminar. Fellows and their sponsors are responsible for their own travel costs to the seminar. The ISCEP supports all other academic and organizational aspects of the seminar.

## Bernard Lown Scholars in Cardiovascular Health Program: Building Capacity to Improve Global Cardiovascular Health

The Lown Scholars Program (LSP) was launched in 2008, thanks to the generous endowment provided by the late Dr Bernard Lown to the Harvard TH Chan School of Public Health. Known for his visionary ideas and his exceptional work in cardiology, Dr Lown had a profound impact on global health, and was recognized with the prestigious Nobel Peace Prize.

Dr Lown fervently believed in the power of knowledge democratization and understood the vital role of local leaders in preventing and managing CVD in LMICs. He entrusted the LSP with the mission of identifying and training these future leaders. The LSP is a testament to his vision and commitment to improving global health.

Since 2008, the Program has been recruiting mid-career public health scientists and professionals through a competitive process, currently supporting 104 Lown scholars from 22 countries across the world. Applicants are required to have a full-time position in a LMIC, and a strong commitment to contribute to improve cardiovascular health both in their contexts and globally.

Each year the Program sponsors about eight to ten new scholars to travel to Boston, where they take a three-week course on Global CVD Prevention designed for them by the Harvard Chan Faculty. They can also take another optional summer course on related topics during this time.

Beyond didactic training, the Program offers robust mentorship. Each new Lown scholar is paired with a Harvard Faculty member and a more experienced Lown scholar, who guide them in developing their research proposals and expanding their collaboration networks. The Program also hosts two monthly online sessions: a townhall meeting to foster a sense of community, and a Discussion Series to keep scholars updated on recent advances in CVD prevention.

An important feature of the Program is providing seed funds for pilot projects. Since 2016, the Program has funded 30 projects, totalling about $800,000. The Program now hosts 14 teams, created around topical or methodological interests and expertise of the scholars to increase the opportunities for collaboration within the Program. Over the years, the journey of building the LSP has been both challenging and rewarding. It has underscored the significance of in-person training and community building, with scholars consistently praising the unique opportunity to immerse themselves in a reach academic environment with their peers.

During its first phase of establishment, the LSP has evolved into a global network of public health scientists and professionals passionate about and with expertise in CVD prevention. Now, as it embarks on its second phase of maturation, the Program is primed for more direct engagement in multi-country interventions for CVD prevention based on scientific evidence generated by our dedicated Scholars.

## World Heart Federation Emerging Leaders Programme (WHFEL): Shaping the Future of Cardiovascular Health

The World Heart Federation Salim Yusuf Emerging Leaders Programme was established in 2014 to develop a long-term cadre of experts who collaborate, research and act to reduce premature mortality from CVD globally. The Programme, developed under the oversight of Professor Salim Yusuf during his term as President of the World Heart Federation, provides training and networking opportunities in cardiovascular health policy and implementation science for healthcare practitioners, researchers and global health advocates.

Each year, after an open, competitive application process, a 25-strong cohort is selected, aiming to be representative by gender and low-middle income settings, and also to have a broad range of skills and experience in cardiovascular health, from clinicians to policymakers and social scientists to ethics and patient organisations.

A central component of the scheme is a bespoke 5-day think tank seminar at different locations each year, focused on a particular area of cardiovascular health (Table 2). Past seminars have included focus on secondary prevention, stroke, digital health, hypertension and the overlap between cardiovascular and infectious diseases. During the seminar, participants receive interactive lectures on implementation science in CVD and are guided by World Heart Federation activities and guidelines or roadmaps in the specific area, so that the ‘wheel is not reinvented’ and outputs align with broader global health efforts.

**Table 2:** World Heart Federation Emerging Leaders Programme-topics and locations of annual seminars.


YEAR	LOCATION	TOPIC

2014	Hamilton, Canada	Secondary prevention

2015	Lima, Peru	Raised blood pressure

2016	Bangalore, India	Tobacco Control

2017	Cape Town, South Africa	Access to medicines

2018	Kunshan, China	Stroke

2019	London, United Kingdom	Heart failure

2020–21 (2020 postponed due to Covid)	Lisbon, Portugal (Hybrid)	Diabetes

2022	Buenos Aires, Argentina	Infectious diseases (Chagas disease, influenza, long covid)

2023	Sydney, Australia	Digital Health (hypertension, atrial fibrillation, coronary artery disease)


The key emphasis during the 5 days of the seminar is to develop a group proposal for a seed-funded implementation project in groups of 8–9 participants (each group receiving funding of US$25,000–30,000) with 2–3 expert mentors. Over the course of the next 12–18 months, the group conducts this project and produces a detailed final report and policy brief (usually culminating in a peer-reviewed scientific publication (Annex)), after regularly updates throughout the timeline. Therefore, each cohort of Emerging Leaders completes three substantive projects. At annual conferences including American College of Cardiology and European Society of Cardiology, satellite meetings have been organised for cohorts of different years to network and develop collaborative projects.

## Discussion – Challenges and Looking to the Future

Across these three global CVD training schemes, there are commonalities in terms of people (including participants and faculty), scope of global CVD research, as well as added value, whether career development, networking, or research impact. The positive impact is supported by participant feedback. In a recent survey of past WHFELs, 92% found the scheme exceptional and 84% felt that they received high-level training in action-oriented research, and feedback from the other schemes is also very positive.

The three complementary schemes are focused on different stages early in the journey of a CVD leader. The ISCEP 10-day seminar is an introductory programme focussed on a general introduction to basic epidemiologic and statistical concepts for prevention research as well as awareness of current issues in CVD epidemiology and prevention, and may represent the starting point for some participants who have gone on to develop careers in CVD prevention research, trials and in policy developments. The WHFEL scheme and the LSP are tailored to individuals already on the CVD career path with more specialised development. It is useful to compare and contrast these three well-established global training programmes for CVD. There are other national and regional CVD capacity-building initiatives, but these three schemes alone have an impressive track record in contributing to the important skills and experience gaps which hold back CVD research, practice and policy.

None of these schemes is without its share of challenges. First, human and financial resources remain the greatest hurdle to scale-up and sustainability of each of these schemes. For example, the ISCEP seminar only continues through identifying hosts in different countries who are willing to identify support for the costs of board and accommodation of the 50 or so participants (40 fellows and 10 faculty) over the 10 days. The main benefit to the host is that the host country has more places allocated for local fellows (n = 6–8). Second, defining the ideal candidates at the ideal time in their career trajectories is important but difficult across countries, professions and contexts, including in terms of ‘early-career’ versus ‘mid-career’. Third, the problem of ‘brain drain’ is real, with participants leaving their home countries soon after the course, looking for better living conditions and career opportunities abroad. Fourth, matching the limited number of places to the greatest need is an ongoing challenge. For example, it is possible for an individual to attend all three of these training and leadership schemes, but should the LMICs with the greatest needs and individuals who have not attended any prior, similar training or courses be prioritised? Finally, these three schemes are all in English language, which is inevitably less accessible to non-English speakers and people who are not fluent in English.

In the digital era, there is more scope for staying in touch with previous participants, networks and for mapping impact, as shown by the monitoring of the favourable LMIC and gender statistics of the different programmes. There is also untapped potential to connect and integrate communities across leadership and training schemes to increase impact of future collaborations and projects. Although informal networking and collaboration has been happening, it is time for more formal collaboration among the network of global cardiovascular researchers, moving forward. There are a growing number of analogous schemes in other diseases and specialties, and there is at least a theoretical threat of vertical training programmes when the challenges which health systems face are across these disease and specialty silos. There is another opportunity and need to map and connect these efforts in the context of ageing populations and multimorbidity.

## Conclusions

There is need, demand and value for global CVD research training programmes for early-career of individuals. There are shared interests and challenges across these three programmes and therefore there are opportunities to learn from one another. The overlap between the programmes further underscores the possibility of better alignment between programmes to improve efficiency and to improve the experience, outputs and impact of individual schemes. There is scope to improve assessment of national and international CVD research needs in order to perhaps check and re-evaluate alignment of such programmes with those needs. These three training programmes, particularly the 10-day seminar, illustrate that even in challenging circumstances, early-career CVD research training can and must be prioritised in the long-term.

## Annex

WHF Emerging Leaders Publications

- Sherifali D, da Silva LP, Dewan P, Cader FA, Dakhil Z, Gyawali B, Klassen S, Yaseen IF, Jovkovic M, Khalid S, Fitzpatrick-Lewis D, Alliston P, Racey M. Peer support for type 2 diabetes management in low- and middle-income countries (LMICs): a scoping review. *Global Heart*. 2024 Feb 20;19(1):20. Doi: 10.5334/gh.1299. PMID: 38404615; PMCID: PMC10885823.
- Isiguzo GC, Santo K, Panda R, Mbau L, Mishra SR, Ugwu CN, Virani SS, Odili AN, Atkins ER. Adherence clubs to improve hypertension management in Nigeria: Clubmeds, a feasibility study. *Global Heart*. 2022;17(1):21. https://doi.org/10.5334/gh.1109.
- Dakhil Z, Yaseen I. The WHF Emerging Leaders Programme: A perspective of two Iraqi Emerging Leaders. 2021.
- Zaidel EJ, Leng X, Adeoye AM, Hakim F, Karmacharya B, Katbeh A, Neubeck L, Partridge S, Perel P, Huffman MD, Cesare MD. Inclusion in the World Health Organization model list of essential medicines of non-vitamin K anticoagulants for treatment of non-valvular atrial fibrillation: a step towards reducing the burden of cardiovascular morbidity and mortality. *Global Heart*. 2020 Aug 6;15(1):52. doi: 10.5334/gh.608. PMID: 32923346; PMCID: PMC7413134.
- Santo K, Isiguzo GC, Atkins E, et al. Adapting a club-based medication delivery strategy to a hypertension context: the CLUBMEDS Study in Nigeria. *BMJ Open*. 2019;9:e029824. doi: 10.1136/bmjopen-2019-029824.
- Huffman MD, Perel P, Eisele JL, Labarthe DR, Wood DA, Sliwa K, Yusuf S. WHF Emerging Leaders Program. *European Heart Journal*. 2017 Oct 1;38(37):2800–2803. doi: 10.1093/eurheartj/ehx483. PMID: 28982234.
- Huffman M, Perel P, Beller G, Keightley L, Miranda J, Ralston J, Reddy K, Wood D, Labarthe D, Yusuf S. World Heart Federation Emerging Leaders Program. *Global Heart*. 2015;10. 10.1016/j.gheart.2014.10.006.

## References

[1] Mensah GA, Fuster V, Murray CJL, Roth GA, Mensah GA, Abate YH, et al. Global Burden of Cardiovascular Diseases and Risks, 1990–2022. J Am Coll Cardiol. 2023 Dec; 82(25):2350–473. 38092509 10.1016/j.jacc.2023.11.007 PMC7615984

[2] Muka T, Imo D, Jaspers L, Colpani V, Chaker L, van der Lee SJ, et al. The global impact of non-communicable diseases on healthcare spending and national income: a systematic review. Eur J Epidemiol. 2015 Apr 18; 30(4):251–77. DOI: 10.1007/s10654-014-9984-2 25595318

[3] Minja NW, Nakagaayi D, Aliku T, Zhang W, Ssinabulya I, Nabaale J, et al. Cardiovascular diseases in Africa in the twenty-first century: Gaps and priorities going forward. Front Cardiovasc Med. 2022 Nov 10; 9. DOI: 10.3389/fcvm.2022.1008335 PMC9686438 36440012

[4] Murphy A, Palafox B, O’Donnell O, Stuckler D, Perel P, AlHabib KF, et al. Inequalities in the use of secondary prevention of cardiovascular disease by socioeconomic status: evidence from the PURE observational study. Lancet Glob Health. 2018 Mar; 6(3):e292–301. DOI: 10.1016/S2214-109X(18)30031-7 29433667 PMC5905400

[5] Filbey L, Zhu JW, D’Angelo F, Thabane L, Khan MS, Lewis E, et al. Improving representativeness in trials: a call to action from the Global Cardiovascular Clinical Trialists Forum. Eur Heart J. 2023 Mar 14; 44(11):921–30. DOI: 10.1093/eurheartj/ehac810 36702610 PMC10226751

[6] Grobbee DE, Pellicia A. Secondary prevention of cardiovascular disease: Unmet medical need, implementation and innovation. Eur J Prev Cardiol. 2017 Jun 16; 24(3_suppl):5–7. DOI: 10.1177/2047487317709369 28618911

[7] Huffman MD, Labarthe DR, Yusuf S. Global Cardiovascular Research Training for Implementation Science, Health Systems Research, and Health Policy Research. J Am Coll Cardiol. 2015 Apr; 65(13):1371–2. DOI: 10.1016/j.jacc.2015.02.023 25835450

[8] Iwelunmor J, Ogedegbe G, Dulli L, Aifah A, Nwaozuru U, Obiezu-Umeh C, et al. Organizational readiness to implement task-strengthening strategy for hypertension management among people living with HIV in Nigeria. Implement Sci Commun. 2023 May 4; 4(1):47. DOI: 10.1186/s43058-023-00425-3 37143131 PMC10157928

[9] Collins D, Laatikainen T, Farrington J. Implementing essential interventions for cardiovascular disease risk management in primary healthcare: lessons from Eastern Europe and Central Asia. BMJ Glob Health. 2020 Feb 12; 5(2):e002111. DOI: 10.1136/bmjgh-2019-002111 PMC7042567 32133194

[10] Weber MB, Baumann AA, Rakhra A, Akwanalo C, Gladys Amaning Adjei K, Andesia J, et al. Global implementation research capacity building to address cardiovascular disease: An assessment of efforts in eight countries. PLOS Global Public Health. 2023 Sep 14; 3(9):e0002237. DOI: 10.1371/journal.pgph.0002237 37708090 PMC10501667

[11] Tudor Hart J. The Inverse Care Law. The Lancet. 1971 Feb; 297(7696):405–12. DOI: 10.1016/S0140-6736(71)92410-X 4100731

[12] Cookson R, Doran T, Asaria M, Gupta I, Mujica FP. The inverse care law re-examined: A global perspective. The Lancet. 2021 Feb; 397(10276):828–38. DOI: 10.1016/S0140-6736(21)00243-9 33640069

[13] Capewell S, Graham H. Will Cardiovascular Disease Prevention Widen Health Inequalities? PLoS Med. 2010 Aug 24; 7(8):e1000320. DOI: 10.1371/journal.pmed.1000320 20811492 PMC2927551

[14] Fuster V, Kelly BB, Vedanthan R. Promoting Global Cardiovascular Health. Circulation. 2011 Apr 19; 123(15):1671–8. DOI: 10.1161/CIRCULATIONAHA.110.009522 21502585

[15] World Heart Federation. Emerging Leaders Programme [Internet]. 2024 [cited 2014 Oct 1]. Available from: https://world-heart-federation.org/emerging-leaders/.

[16] Mensah GA. Training in Cardiovascular Epidemiology and Prevention. Glob Heart. 2018; 13(4). DOI: 10.1016/j.gheart.2018.09.526 30509551

[17] International Society for Cardiovascular Epidemiology and Prevention. Ten Day International Teaching Seminar on Cardiovascular Epidemiology and Prevention [Internet]. 2024 [cited 2014 Oct 1]. Available from: https://iscep.info/seminars.

[18] Harvard TH Chan School of Public Health. The Bernard Lown Scholars in Cardiovascular Health Program [Internet]. 2024 [cited 2014 Oct 1]. Available from: https://www.hsph.harvard.edu/lownscholars/.

